# Carbonic Anhydrase II Activators in Osteopetrosis Treatment: A Review

**DOI:** 10.3390/cimb45020089

**Published:** 2023-02-06

**Authors:** Zikra Alkhayal, Zakia Shinwari, Ameera Gaafar, Ayodele Alaiya

**Affiliations:** 1 Therapeutics & Biomarker Discovery for Clinical Applications, Stem Cell & Tissue Re-Engineering Program, King Faisal Specialist Hospital and Research Centre, P.O. Box 3354, Riyadh 11211, Saudi Arabia; 2 Department of Dentistry, King Faisal Specialist Hospital & Research Center, P.O. Box 3354, Riyadh 11211, Saudi Arabia

**Keywords:** osteopetrosis, carbonic anhydrase II activators, dental abnormalities, osteoclasts, azole, fluconazole

## Abstract

Osteopetrosis is a rare hereditary illness generated by failure in osteoclasts resulting in elevated bone densities. Patients with osteopetrosis possess several complications, like dental caries, earlier teeth loss, delayed eruption, malformed crowns and roots, and lamina dura thickening. Since deficiency of carbonic anhydrase II is a major cause behind osteopetrosis, carbonic anhydrase II activators have a large number of applications in osteopetrosis treatment. There is a lack of a comprehensive review on osteopetrosis, pathogenesis of dental abnormalities, and the role of carbonic anhydrase II activators in osteopetrosis treatment. To address this research gap, the authros perfomed a comprehensive review on osteopetrosis and its types, pathogenesis of dental abnormalities, and the role of carbonic anhydrase II activators in osteopetrosis treatment. A brief introduction to the pathogenesis of dental abnormalities and regeneration is provided in this survey. A discussion of types of osteopetrosis depending on genetic inheritance, such as autosomal dominant, autosomal recessive, and X-linked inheritance osteopetrosis, is presented in this survey. The paper also focuses on the importance of carbonic anhydrase II activators as a potential drug therapy for dental osteopetrosis. In addition, a brief note on the role of azole and fluconazole in treating osteopetrosis is given. Finally, future directions involving gene therapy for dental osteopetrosis are described.

## 1. Introduction

**Figure 1:**
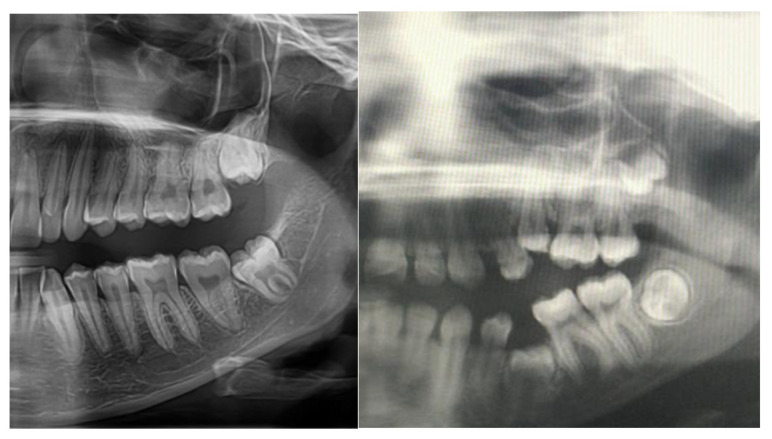
Panoramic radiographs illustrating the teeth of a healthy individual and an individual with osteopetrosis.

Osteopetrosis (OP), commonly known as “marble bone disease”, is a set of uncommon heterogeneous genetic illnesses where the osteoclasts do not function properly, causing the bones to become brittle, thick, sclerotic, and weak. The German radiologist Albers-Schonberg initially identified the disease in 1904, which is often characterized by high bone density on radiographs [1]. On radiographs, the predominant symptom is increased bone density, which is caused by poor osteoclast activity, which also causes reduced bone resorption. Larger and thicker bones are the outcome [2]. The danger of fracture and infection is heightened because of the bones’ increased brittleness. Extreme heterogeneity is caused by at least nine separate forms, each with a unique mechanism of inheritance and severity ranging from asymptomatic to lethal. The dental pulp of human teeth contains mesenchymal stem cells (MSCs), which support the growth or regeneration of teeth. It has been shown that primary deciduous exfoliating teeth’s dental pulp is an important source of stem cells or progenitor cells because of the more varied populations of progenitor/stem cells, like “SHED-stem cells from human exfoliated deciduous teeth”, that live there [3]. It was found during cell characterization that SHED possesses support functions for various markers related to embryonic stem cells. Figure 1 depicts normal and osteopetrosis-affected teeth. Increased osteoclastic activity leading to osteomyelitis in the jaws is a characteristic of the congenital condition osteopetrosis. Unerupted, deformed, or decayed teeth and a high incidence of dental caries because of osteomyelitis and vulnerable dentin and enamel are common orofacial features in individuals with osteopetrosis.

**Figure 2:**
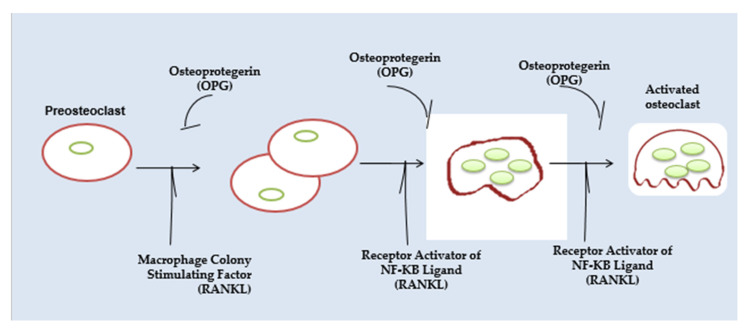
Multinucleated osteoclasts differentiating and becoming active.

Osteopetrosis is a rare hereditary condition marked by an increase in bone mass brought on by impaired osteoclast activity. In recent decades, reports of carbonic anhydrase II genetic abnormalities have been made. These deficiencies have a variety of related phenotypic characteristics, a few of which could result in catastrophic illnesses like osteopetrosis. It is theoretically possible to cure osteopetrosis with carbonic anhydrase II activators. Nevertheless, activators are not used often in the management of osteopetrosis. This is likely because these illnesses are uncommon, which is a good thing, but since they are also complicated medical conditions, it is difficult to detect an inevitable conclusion in clinical trials [4]. Up until recently, functional osteoclast abnormalities were considered the only factor contributing to osteopetrosis in humans. Although the cause of this is unknown, most mutations that impact the production of osteoclasts in humans are probably embryonically fatal [5]. The mutations in genes that encode enzymes involved in the acidic erosion process in the lacuna between the bone matrix and osteoclasts account for the vast majority of osteopetrosis cases. Bone is a tough organ, yet it is continually changing. The adult skeleton has 1–2 million places where the bone is broken down and rebuilt. The osteoclast and the osteoblast are the two key participants in this process [6]. Mesenchymal-derived osteoblasts create the bone’s extracellular matrix, which consists of 90% collagen I. Figure 2 demonstrates the multinucleated osteoclasts differentiating and becoming active.

Bone development, tooth emergence, healing of the bone fracture, and calcium level maintenance in the blood are only a few skeleton-related processes that depend on bone resorption. The main bone-resorbing cells, or osteoclasts, are big, multinucleated cells that are sparsely distributed over the endosteal bone surface [7]. It is now well acknowledged that osteoclasts and macrophages in the BM have a similar progenitor and are both members of the hematopoietic family. These progenitors leave the bloodstream, enter the bone resorption site, and subsequently merge to produce massive, multinucleated cells to become osteoclasts [8]. It is very important to establish the use of carbonic anhydrase II activators in osteopetrosis. There needs to be more literature available establishing the relationship between carbonic anhydrase II activators and osteopetrosis. Therefore, the purpose of this review was to evaluate existing concepts of using carbonic anhydrase II activators in osteopetrosis.

## 2. Review

The review has been divided into six sections, including carbonic anhydrase II activators; osteopetrosis; types of osteopetrosis; pathogens of dental abnormalities and dental pulp stem cells (DPSCS); the role of azole and fluconazole in osteopetrosis; and development of gene therapy for osteopetrosis.

### 2.1. Carbonic Anhydrase II Activators

**Figure 3:**
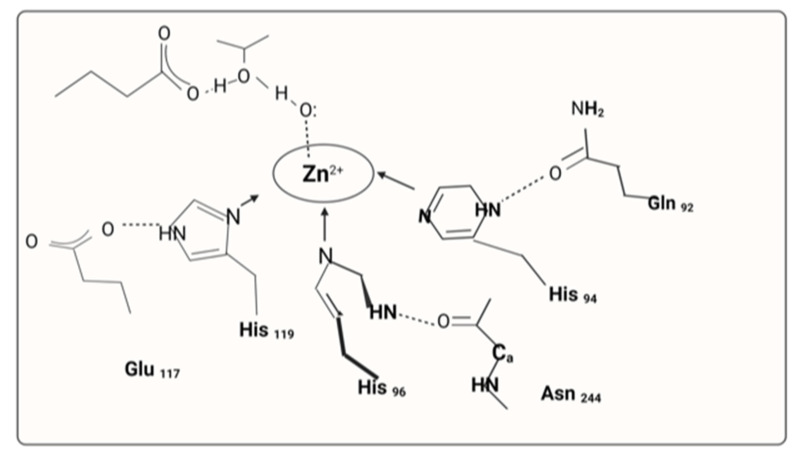
Structure elucidation of carbonic anhydrase II activators.

Carbonic anhydrases II are a class of metalloenzymes that predominantly utilize a metallic hydroxyl carbonyl group reaction to catalyze the reaction between carbon dioxide and bicarbonate. Because they catalyze the rate of hydration of carbon dioxide, carbonic anhydrases II are involved in several metabolic activities related to aerobic respiration and CO_2_ and bicarbonate ion transport via metabolizing tissues, bones, and the lungs. They also regulate several biosynthetic systems such as glycolysis, osteoporosis, lipid metabolism, ureagenesis, bone formation, and mineralization, in addition to managing pH, CO_2_ regulation, electrolyte excretion in many tissues and organs, and other physiological processes [9]. The link between the activator and the enzyme at the catalytic site results in the formation of an enzyme–activator complex [10]. One of the sixteen human α carbonic anhydrase varieties is called carbonic anhydrase II (gene name CA2). Carbon dioxide is reversibly hydrated by carbonic anhydrase. Renal tubular acidosis and osteopetrosis are linked to defects in this enzyme (Figure 3).

Research on reactions has revealed that the catalyst constant (K_c_), not the relationsip between the enzyme and the substrates, is the sole parameter that the activator influences (K_m_) [11]. It was discovered that the electromagnetic spectrum of the ionic interaction between the activator and carbonic anhydrase was identical to that of the isolated enzymes, proving that the activator does not react with the particle itself but rather with a different place [12]. According to cross-crystallographic data, Histamine64 (His64), a key protein for its proton-shuttling function and ability to prevent osteoporosis, was located in the active core of histamine carbonic anhydrase II in two different configurations, far from the Zn ion. The first configuration is known as the “towards” configuration [13] because His64 moves in the direction of the particle [14]. The second configuration is known as the “outer” configuration because His64 moves away from the catalytic site [15]. Studies using cross-crystallography and histamine further show that carbonic anhydrase II activators, which link to the Zn (II), connect far from the particle, near the tooth pulp. Furthermore, the location of His64 is not similar to the location of the active site of the activator [16]. The carbonic anhydrase II activator can be connected to Gln92, to hydrogen bonds with functional groups of Asn62, Asn67, and Gln92, as well as with the water molecule [17]. This results in a rearrangement of the H-bond structure at the catalytic site. By serving as an additional proton shuttle for His64 in the enzymatic primary cycle stage, the activator is primed to participate in this way. This is possible via the interaction between the activator chemical and His64 [18].

### 2.2. Osteopetrosis

Osteoclast function, osteoclast differentiation, and the processes behind osteopetrosis in humans have been well understood thanks to both spontaneous and mutant animal models of the disease [19]. More than twenty distinct mutations have all been identified in mice, and they are all recessive. Table 1 summarizes the three classes of flaws that may be found in these models: polarization and fusion defects, functional defects, and differentiation defects.

Multiple classifications of human osteopetrosis subtypes based on inheritance pattern and severity are available in the scientific literature [20]. The osteopetrosis shown here may be classified into two categories: autosomal dominant and autosomal recessive [21]. The autosomal recessive form may be further divided into two types: a somewhat dangerous type brought on by defects in three genes involved in the reaction and an innocuous variant due to mutations in the gene that encodes CAII (CA2) [22]. Although only a small number of young individuals have been diagnosed with the newly identified severe osteoclast-poor osteopetrosis, these cases will be separated for further study [23].

#### 2.2.1. Osteoclast Function

As osteoclasts cycle through the process of degrading bone, they come with two transfer functions: motile and resorptive [24]. The osteoclast flattens and travels about during the motile stage by developing membrane protrusions known as lamellipodia. Osteoclasts undergo distinctive cytoskeleton reorganization at the point of disintegration as a result of adhesion to the marrow, which polarizes the membrane and creates the seal zone [25]. The actin ring that surrounds the ruffles boundary, known as the sealant zone, separates the resorptive zone from the extracellular space. Avb3 integrins, also known as vitronectin receptors, are present in the sealing zone’s plasma membrane and help to tighten the connection to the underlying bone. Certain proteins in the bone matrix, like sialoprotein and osteopontin, include an amino acid pattern called “Arg-Gly-Asp (RGD)”, with which this matrix receptor interacts [26]. A function for avb3 in resorption has been shown in in vivo investigations using RGD peptides and antibodies targeted against this complex. Mice that lack the b3 subunit of the vitronectin receptor may create osteoclasts, but over time, due to osteoclast malfunction, they develop osteopetrotic bones. The osteoclast’s resorptive organelle, the ruffled border, is created when intracellular acidic vesicles are transported and fused with the patch of plasma membrane encircled by the sealing zone [27]. This procedure brings H^+^–ATPases that transport protons to the ruffled border. These pumps secrete HCl to the resorption region because they are connected to chloride channels [28].

#### 2.2.2. Types of Osteopetrosis

Osteopetrosis is divided into three types and inherited in three different ways: autosomal recessive, autosomal dominant, and X-linked. The most severe type is autosomal recessive osteopetrosis (ARO), with several variations of intermediate severity (IRO) [29]. ARO has a prevalence of 1:250,000 in the general population but is more common in other ethnic groups, particularly Costa Ricans, where the prevalence is substantially greater than elsewhere (3.4:100,000) [30]. IRO remains a less widespread variant, with just a small number of instances reported globally [31].

##### Lack of Carbonic Anhydrase II (CAII) Causes Autosomal Recessive Osteopetrosis

**Figure 4:**
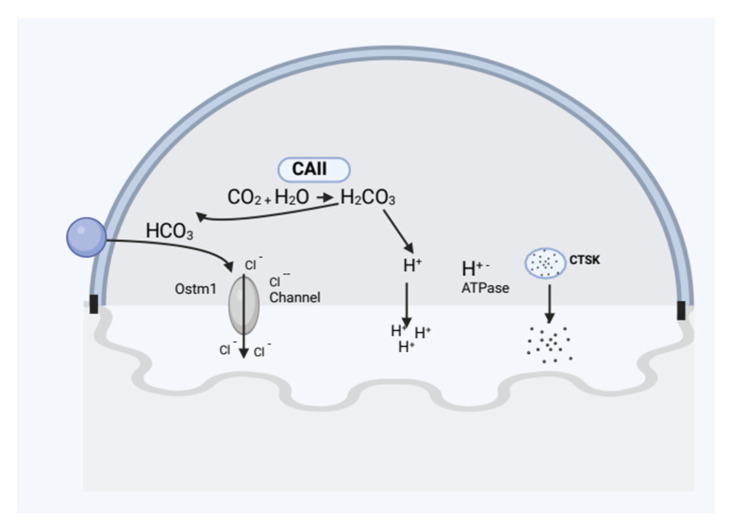
Illustrating the proteins involved in the pathophysiology of human osteopetrosis.

A very mild variation of osteopetrosis that was shown to be brought on by a faulty CAII gene was the first to be identified [32]. One of the CA isoenzymes, CAII, catalyzes the reversible generation of protons in the cytoplasm in osteoclasts. Vacuolar proton pumps then move these protons to the region of resorption. Osteopetrosis symptoms include a predisposition to fractures, dental abnormalities, and issues with vision and hearing that appear during the first 18 months of life [33]. Some individuals see an improvement in their osteopetrotic phenotype as they age. These individuals exhibit additional clinical characteristics because CAII is expressed in various organs [34]. Renal tubular acidosis, caused by CAII’s involvement in HCO_3_ reabsorption in the kidney’s proximal tubule, is the predominant symptom in addition to osteopetrosis [35]. Only a small percentage of people with autosomal recessive osteopetrosis have CAII abnormality. A gene associated with osteopetrosis leads the body to produce too few or aberrant osteoclasts. In the absence of osteoclasts, old bone is not destroyed when new bone is formed, resulting in thick, frail bones. The kind of osteopetrosis a person develops is determined in part by their inherited patterns. Figure 4 represents the proteins involved in the pathophysiology of human osteopetrosis.

##### Osteopetrosis with Autosomal Dominance

The dominant type of osteopetrosis (ADO) is less severe than the recessive forms, and more people are asymptomatic; the prevalence of patients with this condition is likely underestimated [36]. ADO I and II were the two subgroups that made up ADO in the past. Since it has been possible to categorize osteopetrosis using genetic and histological data, suggestions have been made to restrict the definition of osteopetrosis to acute osteoclast deficiencies. It was proposed that this variation be eliminated from the osteopetrosis family of diseases after it was shown that the “increase in bone density in ADO I patients was caused by an osteoblastic defect [37], a mutation in low-density lipoprotein receptor-related protein 5 (LRP5)”. In the process of skeletal growth and remodeling, osteoblasts’ main function is to lay down new bone. Osteoblasts, osteocytes, and hematopoietic stem cells interact directly with other bone cell types throughout this process. A gene associated with osteopetrosis makes too few or faulty osteoclasts, which are bone-resorbing cells. Old bone is not destroyed; new bone is formed when the osteoclasts are absent, resulting in thick, frail bones. One gene, CLCN7, has been identified as the sole source of the genetic abnormality causing ADO II so far. Despite having heterozygous CLCN7 mutations [38], only 2/3 of people with these deficits experience symptoms [39]. The altered protein might primarily harm the channel’s functionality [40], but chloride channels mostly likely exist as a dimer. Long bone fractures are the most typical sign of ADO II, and patients will probably get worse over time.

##### X-Linked Cases (XLO)

Osteopetrosis typically runs in families and is gender-neutral. However, a few X-linked cases (XLO) with lymphedema, infertility, and taking into account dysplasia have recently been documented.

### 2.3. Pathogenesis of Dental Abnormalities

Pathogenesis is the process through which the tooth surface typically loses some tooth minerals due to the acid produced by plaque bacteria after meals containing fermentable carbohydrates are consumed. Saliva generally replenishes this mineral between meals [41]. Giant multinucleated cells called osteoclasts are in charge of breaking down bone. They are created by fusing mononuclear precursor cells, which go to the site of resorption through circulation. Their differentiation is regulated by substances produced by stromal cells in the bone marrow or located on mature osteoblasts. These include RANKL and OPG, to name two [42]. On the surface of the progenitor, the osteoclast is where RANK, the RANKL receptor, is located. As a result, for RANKL and RANK to interact directly to cause osteoclast development and activation, physical contact between the osteoblast or stromal cells and the progenitor osteoclast is required. As an imitation receptor that can attach to RANKL and prevent it from binding to RANK receptors, OPG prevents the production of osteoclasts. As a result, osteoclastogenesis is significantly influenced by RANKL and OPG [43,44]. The blood vessels and nerves found within the tooth might become exposed to bacterial infections causing dental decay if the carious process is allowed to continue. The bacteria start to invade the pulpal system and finally cause pulpal necrosis. A periapical abscess will eventually develop as a consequence of the bacteria-produced toxins starting to leak out via the apical foramen [45]. The maxillary lateral incisor (62.3%) and the mandibular second premolars (60.6%) were the teeth that were missing most commonly. The incisors in the maxilla (97%) and the first premolars in the mandible (43) were the most common supernumerary teeth [46]. Dental abnormalities, including root resorption and early tooth loss, are linked to diseases where osteoclast development or activity is enhanced. Examples include the well-known expansive osteolysis and Paget’s disease. Due to the lack of rodent models for these illnesses as of yet, little is known about the cause of the oral issues in these ailments. The genes that are now known to be mutated in human osteoclast illnesses will be discussed in this brief overview, along with any information on the impact of osteoclast dysfunction on dental development. Genetic dental anomalies are difficulties, disorders, and illnesses of the oral tissues and dentition brought on by faulty genes. Numerous inherited features and deficiencies as well as spontaneous genetic mutations are connected to many hereditary dental and oral abnormalities, many of which are symptoms of more severe illnesses [47].

### 2.4. Dental Pulp Stem Cells (DPSCs)

**Figure 5:**
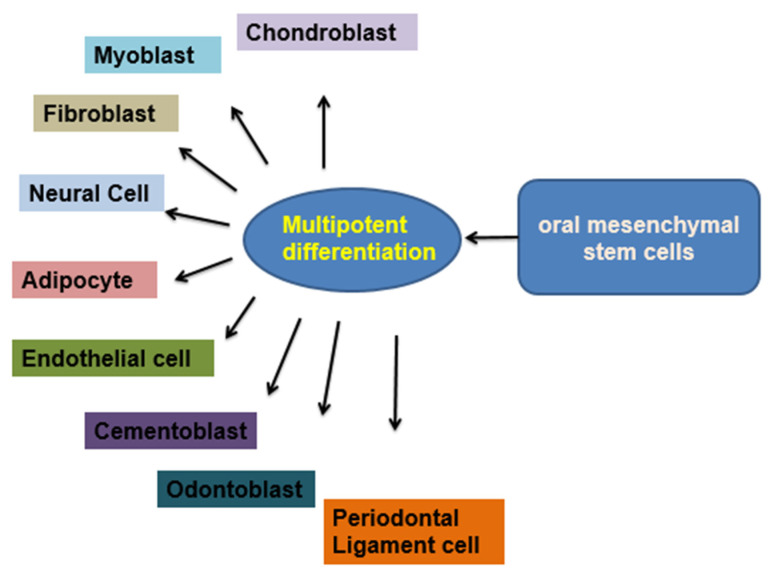
Potential for differentiation of oral mesenchymal stem cells.

The majority of dental anomalies are a result of both environmental and genetic factors. It is possible to notice delayed tooth eruption, missing teeth, deformed teeth that have not yet erupted, hypoplasia of the enamel, periodontal membrane abnormalities [48], malformed crowns and roots, and constriction of the dental pulp chambers. A reduced vascular supply after tooth extraction might lead to osteomyelitis [49]. Due to increasing bone density in osteopetrosis, the dental pulp cells are not easily identifiable. Individuals with osteopetrosis may tend to have bone necrosis caused by destroying the marrow cavities, dental pulp chambers, and tissues and restricting the canals harboring the brachial plexus bundles supplying the jaws and teeth [50]. The crystalline dental pulp, which resides in the pulp chamber at every tooth’s core, comprises soft fibrous tissue, vascular and capillary elements, and neural elements [51]. Endothelial cells, neurons, fibroblasts, osteoblasts/osteoclasts, and odontoblasts are only a few of the cell types that may be found in the dental pulp. hDPSCs are stem cells that originate from the ectoderm and migrate through neural crest cells [52], with a fibronectin structure and other properties of stem cells, adhesion to a solid substrate, and the capacity to differentiate into various cell types. These cells may differentiate into neural stem cells, chondrocytes, adipocytes, odontoblasts, and other cell types under the proper inducing conditions (proliferative rates, colony formation capacity, clonogenic potential, and mineralization potential of DPSCs and BMMSCs). It is claimed that, when compared to BMMSC, DPSCs had a faster rate of proliferating, more clonogenic potential, a larger population of stem/progenitor cells, and maybe more mineralization potential. Additionally, CD29, CD44, CD59, CD73, CD90, and CD146 are mesenchymal markers expressed by dental pulp stem cells. These cells do not express the hematopoietic markers CD34, CD45, or CD11b. The utilization of DPSCs in pancreatic, cardiac, and corneal studies has shown their promise in the domains of regenerative medicine. DPSCs may be distinguished from ICAs, which resemble pancreatic islet cells. The dithizone-positive staining, “C-peptide, PAX4, PAX6, NGN3, and Isl-1-expressing” DPSC-derived ICAs have also shown insulin release in a glucose-dependent pattern to support in vitro functioning. These preliminary findings point to DPSCs as a viable diabetes therapy option in the future. There is a possibility of mending the myocardial infarction (MI) inflicted on naked rats by the secretions of DPSCs of proangiogenic and anti-apoptotic substances. After being injected intramyocardially for four weeks, DPSCs were cultured in vitro and improved cardiac functions. The potential of DPSCs is as an alternate cell population for repairing the heart after an acute MI. In vitro comparisons between DPSCs and limbal stem cells (LSC) demonstrated similarities between the two types of cells, suggesting DPSCs may be a viable alternate source for corneal restoration in patients with unilateral or bilateral complete limb stem cell deficiencies [53,54]. The effective implantation of a “tissue-engineered DPSCs sheet” can restore the epithelium of the cornea in an animal model with fewer limbal stem cells. These results demonstrate the potential of DPSCs in regenerative medicine and provide insights into their unrealized potential. Figure 5 shows the diversity of cell types that may be derived from oral mesenchymal stem cells (MSCs), including odontoblasts, osteoblasts, neural cells, chondrocytes, adipocytes, myoblasts, fibroblasts, and endothelial cells [47].

### 2.5. Role of Azole and Flucanozole in Osteopetrosis

It has been discovered that the V-ATPase at the interface of bone and osteoclast has a unique mix of subunit isoforms. It has been suggested that V-ATPase in multinuclear osteoclasts in osteopetrosis with a greater resorptive ability is overexpressed [54]. Mutations impair V-ATPase activity in osteoclasts in the TCIRG1 gene, which codes for the subunit isoform of V-ATPase. This prevents it from generating protons to the resorption lacunae, which impedes normal bone resorption and regeneration [55]. These mutations result in “infantile malignant autosomal recessive osteopetrosis”, characterized by increased bone density, decreased bone strength, and many skeletal abnormalities. Meanwhile, off-target inhibition of V-ATPase is harmful to the body, and selectivity should be the guiding principle in the creation of therapeutic V-ATPase inhibitors in osteopetrosis. Fluconazole, belonging to the azole family, has been shown to block V-ATPase in osteopetrotic patients. Fluconazole may be administered to individuals with ostepetrosis as an antifungal preventative [56]. Azole as a V-ATPase inhibitor has not yet entered clinical trials, despite the attempts of the scientific community. As a result, its clinical application is quite restricted in V-ATPase targeting in osteopetrosis [57]. Therefore, logical approaches that depend on targeting specific isoforms and V-ATPases present in particular settings known to be related to osteopetrosis by azole must be used. Treatment options for osteopetrosis must include azole treatments that target V-ATPase in the future.

### 2.6. Development of Gene Therapy for Osteopetrosis

One of the most apparent uses of gene therapy is replacing a missing or damaged protein, and inherited monogenic illnesses offer considerable promise as genetic alteration targets [58]. Nearly 100 studies of various monogenic diseases have been conducted throughout the years, the most common being severe combined immunodeficiency (SCID) and Crohn’s disease. The hematopoietic system is a good candidate for gene modification, since it has several favorable characteristics. The ability to access and remove hematopoietic stem cells (HSC) from the body makes it feasible to cultivate and work with them in vitro. Years of clinical expertise from the transplantation of hematopoietic cells are constructive since they may be re-infused into the patient [59]. The science of gene therapy has only had sporadic success up until the late 1990s. Nevertheless, since 2000, many studies have shown the ability of gene therapy that targets HSC to treat X-linked SCID, SCID brought on by an adenosine deaminase defect, as well as chronic granulomatous illness [60]. Although some of these studies had adverse effects, it was evident from them that gene therapy might be the preferred course of treatment for carefully chosen individuals with hematopoietic-system-related monogenic illnesses. The major curative treatment for IMO is this; however, as was already mentioned, when HLA-identical donors are unavailable, this therapy is linked to significant mortality. Since IMO has a fatal result early in childhood when HSCT cannot be used as a treatment, it is a potential illness for gene therapy research. As the condition advances quickly, starting therapy as soon as practicable is critical. Since autologous cells may be used with gene therapy, finding a qualified donor is no longer necessary.

The HSC, with its potential to produce all kinds of mature blood cells, including osteoclasts for both cell therapy, is the chosen cell. This kind of osteopetrosis seems resistant to treatment with HSCT therapy, according to early “BM and spleen cell transplantation trials utilizing the oc/oc mice model”. Later analysis of the oc/oc mice model showed that a loss in the tcirg1 gene was the root cause. It is reasonable to suppose that the mouse variant would be responsive to this kind of treatment, as individuals with a comparable form of human osteopetrosis may be cured with HSCT. This was shown by recent research using the oc/oc model, which showed that both in-utero and neonatal HSCT might effectively treat the majority of the disease’s symptoms. The gene therapy that targets HSC was created using the same animal model after the successful transplantation trials in oc/oc mice. Hematopoietic tissues in radiation-treated neonatal oc/oc mice are being implanted onto genetically modified mice using a retroviral vector to create the tcirg1 and a reporter gene. The results have previously been announced. As a result, about 50% of the mice that received treatment lived. According to the earlier newborn HSCT trial, a level of engraftment of around 20% gene-corrected cells was enough to encourage long-term survival. Both the quantity and appearance of osteoclasts produced during in vitro differentiation of bone marrow cells from treated animals were typical. Nonetheless, compared to osteoclasts generated from bone marrow cells of the wild type, the ability of these cells to resorb bone was only around 10%. Intriguingly, long-lived animals showed virtually full skeletal phenotype normalization as determined by histology and X-ray, despite the fact that a relatively modest degree of osteoclast function correction was demonstrated in vitro.

In the oc/oc paradigm, reversal of the phenotype after gene therapy was much slower than in an experiment wherein neonatal mice received transplants of healthy marrow cells. This suggests that a tiny part of osteoclasts’ ability to resorb bone is required to maintain the equilibrium between bone creation and destruction throughout time. The adult osteoclasts’ low amounts of matrix transgenes could explain why skeletal structure takes longer to normalize after transplant than it does after receiving normal BM. First of its kind, this in vivo investigation of gene repair for osteopetrosis used a vehicle containing a transgene controlled by spleen concentrate creating viral activator, which often continues to expand for most types of cells. To precisely stimulate high expression levels in mature bone resorption and, preferably, a more considerable degree of functional repair, vectors with bone resorption promoters will be needed in the future. By including tcirg1-specific epigenetic marks in the vector, it may be possible to preserve the physical response of signals essential to regulating osteoclast activity. Additionally, it is important to improve osteoclast activity right away after the transplantation of gene-corrected cells. In addition to better viral vectors, approaches to achieve this goal may include differentiating some transduced cells toward the osteoclastic lineage before transplantation or administering an osteoclast-stimulating cytokine, such as RANK-ligand, to transplanted animals [61,62].

This will take extensive monitoring of the transplanted animals and maybe more transplantations. The goal is to demonstrate unambiguously that stem-cell-specific gene treatment in oc/oc mice can completely reverse the condition. It is critical to bear in mind the injection of a significant quantity of stem cells, the requirement for myeloablation, and the longer treatment duration needed to treat OC/OC animals when extrapolating these findings to the human situation. Since afflicted children have smaller BM cavities due to the shrinkage of the marrow space, obtaining enough BM cells for in vitro transduction might be a challenge for gene therapy in people. However, considering that “IMO patients have higher concentrations of stem and progenitor cells in their peripheral blood (PB), one strategy would be to collect PB CD34+ cells for in vitro transduction, maybe even without the requirement for any previous mobilization program”. Only around half of human instances of autosomal recessive osteopetrosis are caused by TCIRG1 abnormalities. HSCT does not reverse other kinds of intrinsic retinal and neural degenerative burdens. This means that molecular identification and utilization of newborn’s HSCT’s potential benefits and limits or future genetic manipulation will need to occur soon after birth in afflicted infants [63].

The heterogeneity of genetic defects resulting in osteoclast dysfunction might cause a variety of clinical variants in osteopetrosis, including malignant and benign diseases [64]. Imaging plays a key role in the diagnosis of such bony diseases [65]. The benign model is a Mendelian-dominant trait, while malignant osteopetrosis is a Mendelian-recessive trait [66]. The malignant type occurs in early childhood and the benign type is commonly reported in individuals in the fourth decade of their life [67]. Currently, the only curative treatment for osteopetrosis is allogeneic hemopoietic stem cell transplantation, and is recommended as early as possible. Despite the disadvantages described above, we consider the restoration of normal bone formation in oc/oc mice as a critical first step in the advancement of gene therapy for people with malignancy osteopetrosis. In addition, this is the first time that genomic targeting of bone resorption has been achieved, providing new avenues for studying and treating diseases by manipulating the actions among these cells.

## 3. Conclusions

The majority of osteoclast abnormalities seen in individuals with osteopetrosis may be reliably replicated in vitro, aiding in diagnosis and significantly advancing preclinical research on the biology of osteoclasts and osteopetrosis. This review has established the role of carbonic anhydrase II activators in osteopetrosis and the need for more data regarding their relationship. Nevertheless, further research is still required to understand better the variables influencing the clinical role of carbonic anhydrase II activators in osteopetrosis.

## Figures and Tables

**Table 1:** Mutations in human osteopetrosis.

Gene	Human Osteopetrosis	Osteoclasts (OC) Features	Protein	Protein Function
**Differentiation defects**				
CSF1	No	The number of OC is less	M-CSF	Cytokine (M-CSF)
CSFIR	No	The number of OC is less	C-FMS	MSC-F receptor
FOS	No	No OC	c-fos	Transcription factor
SPI1	NO	No OC	PU.1	Transcription factor
TNFRSF11A	No	No OC	RANK	RANKL receptor
TNFSF11	ARO	No OC	RANKL	Cytokine
**Fusion and polarization defects**				
MITF	No	The number of OC is normal and no RB	MITF	Transcription factor
NFKB1/NFKB2	No	The number of OC is less	NF-kB1/2	Transcription factor
TRAF6	No	The number of OC is normal and no RB	TRAF6	Adaptor protein
TYROBP	No	The number of OC is less	DAP12	Adaptor protein
**Functional defects**				
ACP5	No	The number of OC is high and no RB	TRACP	Acid phosphatase
CLCN7	ARO ADO	OC is enlarged with less developed RB	CLCN7	Chloride channel
CTSK	Pycnodystosis	The number of OC is normal with irregular RB	Cathepsin K	Cathepsin
ITGBI	No	The number of OC is high and no RB	Itg$\beta_{3}$	Integrin
OSTM1	ARO	The number of OC is high with underdeveloped RB, and deformed cytoskeleton	OSTM1	Subunit for CLCN7?
SRC	No	The number of OC is high and no RB	c-src	Tyrosine kinase
TCIRGI	ARO	The number of OC is high and no RB	TCIRGI	$H^{+}–\mathrm{ATPase}$

## Data Availability

Not applicable.
